# Data invisibility in United States national healthcare datasets: implications for immigrant health equity

**DOI:** 10.1016/j.lana.2026.101574

**Published:** 2026-07-18

**Authors:** Tala Al-Rousan, Matthew Banegas, Ava Nariman, Cheryl A.M. Anderson

**Affiliations:** a Herbert Wertheim School of Public Health and Human Longevity Science, University of California, La Jolla, San Diego, CA, USA; b Department of Radiation Medicine and Applied Sciences, University of California, San Diego, La Jolla, San Diego, CA, USA

**Keywords:** Immigrant health, Health surveillance, Data invisibility, Health equity, Immigration policy

## Abstract

Immigrants represent more than 47 million people in the United States yet remain systematically invisible in the national datasets that inform health policy. Data invisibility operates across three dimensions: the structural absence of immigration related variables from most major national datasets; the substitution of imprecise proxy indicators that introduce misclassification bias; and the deliberate withdrawal of immigrant populations from data systems in response to enforcement risk. While some federal health surveys collect basic nativity data, these measures do not capture the full complexity of immigration history; recent federal arrangements permitting the use of health and tax data for immigration enforcement have compounded both the practical and ethical barriers to expanded data collection. International examples from Denmark, Sweden, and Canada demonstrate that comprehensive, ethically governed migration data collection is operationally feasible. We argue that expanded data collection may be epidemiologically necessary but remains ethically contingent upon legal protections, community governance, and restoration of trust.

## Immigrant health disparities and data invisibility

Immigrants comprise a substantial and growing share of the United States (US) population, totaling more than 47 million individuals and accounting for up to 14% of the rural workforce.^1^ Despite vital contributions to the healthcare, agriculture, construction, and science, technology, engineering and math (STEM) sectors, immigrants face persistent care access barriers, with lower healthcare utilization and spending compared with US-born populations.^2^^,^^3^ Although literature suggests a “healthy immigrant effect” to explain this pattern, this concept is increasingly being challenged, refined and qualified by researchers, underscoring the need for more research.^4^^,^^5^ Immigrant health disparities are substantially driven by structural factors: restricted insurance eligibility, exclusion from public programs, and historically grounded mistrust of formal health systems shaped by institutional discrimination.^6^^,^^7^ A central obstacle to understanding, monitoring, and addressing these inequalities is data invisibility—the systematic absence of direct immigration-related variables from the major administrative and survey datasets that support US population health research.^7^

A proposal to expand immigration-related data collection must, however, be understood within the political context in which it emerges. In April 2025, the Internal Revenue Service (IRS) formalized an information-sharing agreement with Immigration and Customs Enforcement (ICE) permitting the use of taxpayer data in deportation operations^8^; concurrently, the Centers for Medicare and Medicaid Services (CMS) transferred Medicaid enrollee data to the Department of Homeland Security (DHS), prompting litigation by twenty state attorney generals and a federal court finding that the policy likely violated the Administrative Procedure Act.^9^ These events demonstrate that immigrant communities’ hesitation to engage with institutions and systems does not stem from irrational distrust, but from observable governmental actions. Research with immigrant patients across multiple US care settings confirms that many have adopted data invisibility as a deliberate coping strategy, in direct response to the realistic threat of enforcement.^10^ Accordingly, this paper does not advocate for the unrestricted expansion of data collection. Rather, it argues that immigration-related data can provide important epidemiologic and policy insights, while emphasizing that the legal, ethical, and social conditions required to justify expanded collection remain contested and incomplete.

## Data gaps and limitations of national datasets

Data invisibility, as used here, encompasses three distinct but related phenomena: structural non-measurement, the absence of immigration-related variables from datasets by design; measurement error, the substitution of proxy indicators that misclassify immigrant status; and behavioural non-participation, the deliberate withdrawal of immigrant populations from data systems in response to enforcement risk. Each dimension compounds the others, and each requires a distinct policy response. The following sections address each in turn, beginning with the structural absence of direct immigration measures across the national datasets that serve US population health research.

Most major national administrative datasets and electronic health record networks lack direct measures of immigration status or history, relying instead on indirect proxy indicators such as surname algorithms, geographic characteristics, or preferred language (Table 1). While NHIS and NHANES do collect nativity and country-of-birth data, and NHIS additionally asks about citizenship status, these measures vary in consistency and do not capture the full complexity of immigration history.^11^, ^12^, ^13^ In datasets relying on such proxies, misclassification bias is common and heterogeneity within immigrant populations is obscured, producing underestimates of health disparities and poorly targeted interventions. The limitations of proxy measures are not merely theoretical. A recent validation study examined a widely used proxy for legal status against one of the largest legalization events in U.S. history; the legalization of approximately two million Mexican immigrants under the Immigration Reform and Control Act (IRCA). The proxy showed little detectable change in legal status, despite administrative evidence that large-scale legalization had occurred; whereas direct measures of legal status tracked the policy change as expected. These findings suggest that proxy measures may be inadequate for evaluating the health consequences of immigration policies or identifying populations affected by policy interventions. The limitations of proxy measures extend beyond statistical imprecision. Many policy-relevant exposures, including migration pathway, duration of residence, refugee status, and legal precarity cannot be inferred reliably from language, surname, or geographic characteristics. As a result, populations with substantially different health risks and barriers to care are often grouped together, limiting the ability of public health agencies to identify vulnerable subgroups, evaluate policy effects, or design targeted interventions. Proxy measures may distinguish immigrants from non-immigrants in broad terms, but they are poorly suited to answering the questions most relevant for advancing immigrant health equity. Moreover, proxy-based approaches do not eliminate ethical concerns; they merely relocate them. Surname algorithms, geographic profiling, and language-based classifications infer immigration-related characteristics without respondents’ explicit participation and may incorrectly assign individuals to immigrant categories. These methods therefore combine reduced accuracy with limited transparency, weakening both scientific validity and accountability.

**Table 1:** Immigration-related variables and proxy indicators in selected US National datasets.

Dataset	Description	Direct immigration variables	Proxy indicators used
Federal health surveys			
National health interview survey (NHIS)	Annual household survey; primary federal source for health status and care access data.	Country of birth; citizenship status; year of immigration	Preferred language; interpreter use; race–ethnicity
National health and nutrition examination survey (NHANES)	National survey combining interviews and physical examinations; primary source for disease and nutrition surveillance.	Country of birth; citizenship status; years of US residence	Language spoken at home; interpreter need
National survey on drug use and health (NSDUH)	Annual survey of substance use and mental health behaviours among the US civilian population.	Country of birth	Race–ethnicity; preferred language
Medical expenditure panel survey (MEPS)	Nationally representative household survey of healthcare use, costs, and insurance coverage; drawn from the NHIS outgoing panel.	Nativity status; English language proficiency	Race–ethnicity; insurance type; income
Administrative & claims data			
American community survey (ACS)	Large national demographic survey; frequently linked to health datasets.	Nativity; citizenship status; year of arrival; English proficiency; ancestry	N/A; direct measures routinely collected
Survey of Income and Program Participation (SIPP)	Census Bureau longitudinal panel survey on economic well-being and program participation	Country of birth; citizenship status; year of entry; permanent legal resident status	Legal status is inferred from lawful permanent resident questions
Medicaid claims data	Administrative claims covering low-income individuals and children.	None	Preferred language; insurance type (e.g., emergency Medicaid); ZIP code; surname algorithms
Medicare claims data	Administrative claims for adults aged ≥ 65 years and certain disability populations.	None	Language preference; surname-based ethnicity estimation; geographic ethnic density
CDC Wide-ranging Online Data for Epidemiologic Research (WONDER)	National mortality and cause-of-death database.	None	Surname algorithms; geographic ethnic composition
EHR networks & cohort studies			
All of us research	Platform used to access surveys, genomic analyses, EHRs, and physical measurements to study the full range of factors that influence health and disease.	Country of birth	Race–ethnicity; preferred language; geographic area
Health and retirement study (HRS)	Longitudinal study of US adults aged ≥ 50 years; focuses on ageing, retirement, and socioeconomic conditions.	Nativity; year of arrival; citizenship status	Language; English proficiency; race–ethnicity; region of birth
OCHIN electronic health record network	Multistate community health centre EHR serving safety-net populations.	None	Preferred language; interpreter need; insurance type; address
COSMOS (Epic electronic health record network)	Large federated EHR data platform aggregating records from Epic health systems nationwide for clinical research and population health analyses.	None	Preferred language; race–ethnicity; insurance type; geographic area
PCORnet network (National patient-centered clinical research network)	National clinical research network integrating EHR and claims data from health systems, academic medical centers, and community health networks across the United States	None	Preferred language; race–ethnicity; insurance type; geographic area

The American Community Survey (ACS) is unusual among major national datasets in the breadth and consistency of its migration-related measures, routinely collecting nativity, citizenship, year of arrival, English proficiency, and ancestry. Although some federal surveys, including NHIS and NHANES, collect selected migration-related variables, these measures are not consistently available across the broader surveillance and administrative data infrastructure that informs population health research. For instance, the primary national mortality database, CDC WONDER, contains no direct migration variables, nor does foundational chronic disease surveillance infrastructure, meaning that cause-of-death data cannot be reliably stratified by immigration history (Table 1).^11^ Large EHR research networks, including OCHIN, PCORnet, and COSMOS, offer substantial opportunities for identifying disparities, yet these systems generally lack standardized immigration-related variables and, therefore, continue to rely on proxy measures. When diverse populations are aggregated under a single label such as “language-spoken,” or “interpreter-use” meaningful variation in health risks, care-seeking behaviours, and structural barriers is overlooked as these labels are not consistently captured across platforms. Clinical proxies such as interpreter use or emergency Medicaid enrollment similarly limit the design of targeted interventions and contribute to resource misallocation.^3^

For example, in cancer surveillance, birthplace data are absent for between 31% and 49% of cases depending on cancer site. Because nativity is frequently estimated through surname-based and related proxy approaches, observed disparities in cancer incidence and survival vary according to how missing nativity is assigned.^14^ Studies using improved methods to recover nativity information have demonstrated that estimates of immigrant–US-born differences can shift substantially, indicating that proxy-based approaches may obscure clinically meaningful disparities and complicate efforts to target screening and prevention programs.^15^^,^^16^

These measurement failures not only determine policy outcomes in isolation, but also political will, resource allocation, and institutional capacity, which mediate the relationship between data and action. Nevertheless, where surveillance infrastructure systematically obscures the health burden of a population, the evidence base required to justify targeted intervention is absent, and policy responses are correspondingly constrained.

## International models for migration data collection

Several countries have demonstrated that the limitations of US health surveillance are not inevitable. Denmark, Sweden, and Canada conceptualize migration as a longitudinal process and maintain centralized, linkable population registries that routinely capture country of birth, parental origin, duration of residence, and specific migration category.^17^, ^18^, ^19^

Denmark’s Civil Registration System links individual and intergenerational migration histories for more than 5.9 million residents to data on health-care utilization, morbidity, and mortality.^19^ Swedish population registries record first- and second-generation migration status alongside duration of residence, enabling analysis of how health risks evolve over time.^17^ Canada’s Longitudinal Immigration Database integrates immigration, census, and health records to support analyses stratified by migration category.^18^ Collectively, these systems address the core failures of US datasets, incomplete coverage, sampling bias, and limited follow-up, while also demonstrating that robust governance frameworks can maintain public trust without sacrificing analytical power.

These models are not directly transferable. Registry-based data centralization raises legitimate concerns about privacy and the potential for state misuse; there concerns have particular salience in the US context. Governance structures in Denmark, Sweden, and Canada differ substantially from the US federal system. Their relevance lies not in direct replication but in demonstrating that comprehensive, ethically governed migration data collection is operationally feasible.

## Ethical preconditions for data reform

The case for expanded immigration data collection in the US must contend with an ethical tension that international models do not resolve. Although immigration-related data can help identify health disparities, inform interventions, and evaluate policies affecting immigrant health, undocumented and mixed-status populations have historically avoided official data systems in the U.S. Past precedents of data misuse, including the use of Census data to facilitate the internment of Japanese Americans during the Second World War and the routine sharing of social program data with immigration authorities prior to established firewall policies, establish that the risks of participation are grounded in historical experience.^20^

These concerns have measurable consequences: the 2025 KFF/New York Times Survey of Immigrants found that concerns about immigration status led 12% of immigrant adults to avoid applying for food, housing, or health-care assistance and 11% to discontinue participation in these programs.^21^ Such avoidance not only limits access to essential services but also produces incomplete and potentially biased datasets that obscure health needs among the populations most vulnerable to exclusion.^4^ Data collection is therefore not an unqualified good; its value depends on whether it can generate meaningful health benefits without creating new risks. The justification for expanded collection rests on two conditions: that affected communities are meaningfully involved in instrument design under independent oversight and that Congressional legislation prohibits the use of federally collected health data for civil immigration enforcement. Even where such protections are enacted, participation may remain limited if immigrant communities do not perceive them as credible; trust is a social and historical condition that cannot be created through legislation alone. Meaningful participation will likely require sustained evidence that collected data are not being used for enforcement purposes and that governance structures operate independently of immigration authorities. Where these conditions are not met, the ethical justification for expanded collection is substantially weakened.

## Conditions for ethical expansion of immigration-related data collection

The most immediately urgent priority is statutory protection. Administrative policy safeguards have proven insufficient and no ethical case for expanded immigration data collection can be made in their absence; the reversal of longstanding IRS and CMS privacy commitments within a single administration demonstrates their fragility.^8^, ^9^, ^10^ Durable protection therefore requires federal legislation that explicitly prohibits the use of federally collected health data for civil immigration enforcement. Technical safeguards, such as de-identification procedures and strict access controls, are important, but they cannot substitute for binding legal protections. Until such statutory protections are in place, immigration-related data collection in federal health surveys and administrative systems should not proceed.

Where the legal foundation exists, the second reform is administratively feasible within existing federal survey infrastructure: standardization of validates, non-stigmatizing migration measures. National datasets, such as the National Survey on Drug Use and Health (NSDUH), the Medical Expenditure Panel Survey (MEPS), and Medicaid and Medicare claims data should adopt validated instruments that capture life-course migration histories, including duration of residence and migration category, through multilingual administration and inclusive sampling strategies. Furthermore, it is crucial to avoid the collection of legal or political details that could be used against respondents.^22^ These standardized measures should replace inaccurate proxy indicators across federal surveillance infrastructure.

The third reform requires long-term investment: development of secure, linkable population registries modelled on the international examples described above.^17^, ^18^, ^19^ Federal agencies should invest in migration variables with health and social data, under strict ethical oversight and independent governance to enable analyses that distinguish migration-related exposures from race, ethnicity, and socioeconomic status. The international models reviewed here demonstrate this is operationally feasible; however, the primary barriers in the US context are legal and political rather than technical.

The fourth reform should be pursued in parallel with the abovementioned: the formal inclusion of immigrant communities in the design of data collection strategies. Community representatives should be active partners in the development of research instruments, sampling approaches, and data governance frameworks to ensure cultural appropriateness, foster trust, and promote meaningful participation. Research conducted on communities rather than with them has poor external validity and limited acceptability.

Standardizing these measures across federal health surveillance infrastructure would transform immigration status from a persistent data gap into an analytical asset. The variation documented in Table 1 is not inevitable; it reflects choices about whose health is considered worth measuring. If unchanged, these choices will allow for 47 million people to remain invisible to health systems.^1^

## Contributors

All authors contributed to the conceptualization of the study. TA and AN drafted the original manuscript. MA and CA authors contributed to reviewing and editing the manuscript. TA provided supervision and managed project administration. All authors reviewed and approved the final version of the manuscript.

## Declaration of interests

We declare no competing interests.
